# Effects of Hearing Intervention on Mobility Disability Over 3 Years: Findings From the ACHIEVE Study

**DOI:** 10.1093/geroni/igaf122.077

**Published:** 2025-12-31

**Authors:** Pablo Martinez-Amezcua, Emmanuel Garcia-Morales, Sarah Bessen, Alison Huang, Jennifer Deal, Nicholas Reed, Jennifer Schrack, Frank Lin

**Affiliations:** Johns Hopkins Bloomberg School of Public Health, Baltimore, Maryland, United States; New York University, New York, New York, United States; Johns Hopkins School of Medicine, Baltimore, Maryland, United States; Johns Hopkins Bloomberg School of Public Health Department of Epidemiology, Baltimore, Maryland, United States; Johns Hopkins Bloomberg School of Public Health, Baltimore, Maryland, United States; New York University Grossman School of Medicine, New York, New York, United States; Johns Hopkins Bloomberg School of Public Health, Baltimore, Maryland, United States; Johns Hopkins Bloomberg School of Public Health, Baltimore, Maryland, United States

## Abstract

Hearing loss is associated with increased functional limitations and difficulties in daily activities. However, whether treating hearing loss slows down the decline in daily life functioning remains unknown. Using data from the Aging Cognitive Health Evaluation in Elders (ACHIEVE) study, a 1:1 randomized control trial of a hearing care intervention (including the provision of hearing aids) compared to a healthy education control group, we investigated the effect hearing care vs. control on daily life functioning, assessed by three domains (functional limitations [e.g., walking ¼ mile, crouching], activities of daily living [ADLS; e.g., getting in and out of bed, dressing], and instrumental ADLS [IADLS; e.g., house chores, preparing meals]), with the Physical Ability Questionnaire, among 977 community-dwelling older adults with hearing loss recruited from the Atherosclerosis Risk in Communities Study (ARIC [n = 238]) and de novo from the community (n = 739). Over three years, there was an increase in the proportion of participants reporting functional limitations (from 65.6% to 73.3%), difficulties in ADLS (from 11.8% to 1838%), and IADLS (from 19.3% to 26.7%). The 3-year change in these proportions did not differ between the hearing intervention arm and the health education control participants. Our findings suggest that compared to a health education control, a hearing intervention does slow down the progression of functional limitations or difficulties with daily (instrumental or not) activities. Further research is warranted to explore additional factors influencing daily function in older adults with hearing loss.

